# Supplementary material for: Calculation of Lipophilicity of Organophosphate Pesticides Using Density Functional Theory
Source: Membranes (Basel). 2022 Jun 19;12(6):632. doi: 10.3390/membranes12060632 (PMC9228327; doi:10.3390/membranes12060632)
Supplement: Supplementary file 1 [file membranes-12-00632-s001.zip › membranes-1747464-supplementary.pdf]

Supplementary Information

The Calculation of Lipophilicity of the Organophosphate Pesticides with Using Density Functional Theory

Magomedov K.E.[1,2,*], Zeynalov R.Z.[2], Suleymanov S.I.[2,3], Tataeva S.D.[2], Magomedova V.S.[2]

1. Immanuel Kant Baltic Federal University, REC "Smart Materials and Biomedical Applications", Aleksandra Nevskogo str., 14, Kaliningrad 236041, Russia; m_kurban@mail.ru
2. Dagestan State University, Department of analytical and pharmaceutical chemistry, 367000, Makhachkala, M. Gadzhieva st., 43a.
3. Analytical Center for Collective Use, Dagestan Federal Research Centre of the Russian Academy of Sciences, 367001, Makhachkala, Russia

* Correspondence: m_kurban@mail.ru

Table S1. IUPAC names of all 22 OPs pesticides investigated in present study

**Pesticide**	**Systematic name**
Acephate	N-[Methoxy(methylsulfanyl)phosphoryl]acetamide
Aspon	O,O,O′,O′–Tetrapropyl dithiopyrophosphate
Carbophenothion	S-[[(4-Chlorophenyl)thio]methyl] O,O-diethyl phosphorodithioate
Chlorpyrifos	O,O-Diethyl O-3,5,6-trichloropyridin-2-yl phosphorothioate
Coumaphos	O-(3-Chloro-4-methyl-2-oxo-2H-chromen-7-yl) O,O-diethyl phosphorothioate
Crufomate	N-[(4-tert-Butyl-2-chlorophenoxy)methoxyphosphoryl]methanamine
Diazinon	O,O-Diethyl O-[2-isopropyl-6-methylpyrimidin-4-yl] phosphorothioate
Dichlorvos	2,2-Dichloroethenyl dimethyl phosphate
Dimethoate	O,O-Dimethyl S-[2-(methylamino)-2-oxoethyl] phosphorodithioate
Dioxathion	S,S'-1,4-Dioxane-2,3-diyl O,O,O',O'-tetraethyl bis(dithiophosphate)
Disulfoton	O,O-Diethyl S-[2-(ethylsulfanyl)ethyl] phosphorodithioate
Ethion	O,O,O′,O′-Tetraethyl S,S′-methylene bis(phosphorodithioate)
Fenitrothion	O,O-Dimethyl O-(3-methyl-4-nitrophenyl)phosphorothioate
Fenthion	O,O-Dimethyl O-[3-methyl-4-(methylsulfanyl)phenyl] phosphorothioate
Fonofos	O-Ethyl S-phenyl ethylphosphonodithioate
Malathion	Diethyl 2-[(dimethoxyphosphorothioyl)sulfanyl]butanedioate
Methyl Parathion	O,O-Dimethyl-O-4-nitrophenylphosphorothioate
Monocrotophos	Dimethyl (E)-1-methyl-2-(methylcarbamoyl)vinyl phosphate
Parathion	O,O-Diethyl O-4-nitrophenyl phosphorothioate
Phorate	O,O-Diethyl S-[(ethylsulfanyl)methyl] phosphorodithioate
Phosalone	6-Chloro-3-{[(diethoxyphosphinothioyl)sulfanyl]methyl}-1,3-benzoxazol-2-one
Temephos	O,O,O′,O′-Tetramethyl O,O′-sulfanediylbis(1,4-phenylene) diphosphorothioate

Table S2. Formulas according to which the statistical processing of the results of quantum-chemical modeling was carried out. For a quantitative value n, if y_i is the estimated value and Y_i is the experimental value, the estimates are calculated as follows:

Equation	No
$ME = \frac{1}{n}\sum_{i=1}^{n}(y_i - Y_i)$	(S1)
$MAD = \frac{1}{n}\sum_{i=1}^{n}\lvert y_i - Y_i\rvert$	(S2)
$MSE = \frac{1}{n}\sum_{i=1}^{n}(y_i - Y_i)^2$	(S3)
$MPE = \frac{100}{n}\sum_{i=1}^{n}\frac{y_i - Y_i}{Y_i}$	(S4)
$MAPE = \frac{100}{n}\sum_{i=1}^{n}\left\lvert\frac{y_i - Y_i}{Y_i}\right\rvert$	(S5)
$SLRL = \frac{\sum_{i=1}^{n}(y_i - \bar{y}_i)(Y_i - \bar{Y}_i)}{\sum_{i=1}^{n}(Y_i - \bar{Y}_i)^2}$	(S6)

$$r = \frac{\sum_{i=1}^{n}(y_i - \bar{y_i})(Y_i - \bar{Y_i})}{\sqrt{\sum_{i=1}^{n}(y_i - \bar{y_i})^2 (Y_i - \bar{Y_i})^2}} \quad (S7)$$

$$PCC = r^2 \quad (S8)$$

Table S3. Calculated solvation free energy change of transfer from the gas phase to water phase (ΔGsolv(water) / kcal mol−1) and octanol phase (ΔGsolv(octanol) / kcal mol−1) under standard state conditions, and corresponding log KOW values of examined OPs pesticide set at B3LYP/DEF2-SVP level of theory, with experimentally determined log P.

OPs	ΔGOctanol	ΔGWater	LogKo/w(PBE-def2-SVP)	LogPexp	Ref.
Acephate	-63.71	-67.90	-0.73	-0.80	[1,2]
Aspon	-64.77	-22.30	7.44	6.00	[1,2]
Carbophenothion	-68.48	-38.65	5.23	5.30	[1,2]
Chlorpyrifos	-47.88	-16.78	5.45	5.00	[1,3]
Coumaphos	-75.65	-56.83	3.30	4.50	[1,4]
Crufomate	-65.48	-52.31	2.31	3.40	[1,2]
Diazinon	-51.50	-27.60	4.19	3.80	[1,2]
Dichlorvos	-34.98	-22.67	2.16	1.40	[1,2]
Dimethoate	-68.81	-66.63	0.38	0.80	[1,2]
Dioxathion	-86.57	-54.62	5.60	4.30	[1,2]
Disulfoton	-57.59	-29.23	4.97	4.00	[1,2]
Ethion	-69.85	-49.66	3.54	5.10	[1,2]
Fenitrothion	-46.26	-31.87	2.52	3.30	[1,2]
Fenthion	-47.26	-23.50	4.16	4.10	[1,5]
Fonofos	-62.34	-39.63	3.98	3.90	[1,2]
Malathion	-59.98	-49.22	1.89	2.40	[1,2]
Methyl Parathion	-43.86	-31.52	2.16	2.90	[1,2]
Monocrotophos	-66.07	-70.97	-0.86	-0.20	[1,2]
Parathion	-49.61	-31.33	3.20	3.80	[1,2]
Phorate	-50.94	-25.34	4.49	3.60	[1,2]
Phosalone	-74.83	-57.01	3.12	4.40	[1,2]
Temephos	-75.58	-41.72	5.94	6.00	[1,2]

Table S4. Calculated solvation free energy change of transfer from the gas phase to water phase (ΔGsolv(water) / kcal mol−1) and octanol phase (ΔGsolv(octanol) / kcal mol−1) under standard state conditions, and corresponding log KOW values of examined OPs pesticide set at PBE/DEF2-SVP level of theory, with experimentally determined log P.

OPs	ΔGOctanol	ΔGWater	LogKo/w(PBE-def2-SVP)	LogPexp	Ref.
Acephate	-60.27	-58.11	-0.38	-0.80	[1,2]
Aspon	-18.39	-61.15	7.49	6.00	[1,2]
Carbophenothion	-36.73	-67.08	5.32	5.30	[1,2]
Chlorpyrifos	-13.10	-42.92	5.23	5.00	[1,3]
Coumaphos	-49.89	-69.94	3.52	4.50	[1,4]
Crufomate	-44.09	-61.05	2.97	3.40	[1,2]
Diazinon	-25.48	-49.70	4.25	3.80	[1,2]
Dichlorvos	-16.70	-30.45	2.41	1.40	[1,2]
Dimethoate	-60.84	-64.91	0.71	0.80	[1,2]
Dioxathion	-47.37	-76.90	5.18	4.30	[1,2]
Disulfoton	-26.74	-56.24	5.17	4.00	[1,2]
Ethion	-45.09	-67.44	3.92	5.10	[1,2]
Fenitrothion	-25.87	-41.28	2.70	3.30	[1,2]
Fenthion	-23.46	-47.25	4.17	4.10	[1,5]
Fonofos	-37.40	-60.55	4.06	3.90	[1,2]
Malathion	-43.06	-55.25	2.14	2.40	[1,2]
Methyl Parathion	-25.70	-38.94	2.32	2.90	[1,2]

Monocrotophos	-62.72	-60.78	-0.34	-0.20	[1,2]
Parathion	-25.55	-45.61	3.52	3.80	[1,2]
Phorate	-26.15	-49.70	4.13	3.60	[1,2]
Phosalone	-48.89	-68.13	3.37	4.40	[1,2]
Temephos	-34.90	-70.76	6.28	6.00	[1,2]

Table S5. Calculated solvation free energy change of transfer from the gas phase to water phase (ΔGsolv(water) / kcal mol−1) and octanol phase (ΔGsolv(octanol) / kcal mol−1) under standard state conditions, and corresponding log KOW values of examined OPs pesticide set at PBE/DEF2-TZVP level of theory, with experimentally determined log P.

OPs	ΔGOctanol	ΔGWater	LogKo/w(PBE-def2-SVP)	LogPexp	Ref.
Acephate	-68.00	-73.43	-0.95	-0.80	[1,2]
Aspon	-69.44	-26.05	7.60	6.00	[1,2]
Carbophenothion	-73.02	-44.02	5.08	5.30	[1,2]
Chlorpyrifos	-49.06	-20.33	5.04	5.00	[1,3]
Coumaphos	-81.19	-63.41	3.12	4.50	[1,4]
Crufomate	-69.99	-54.07	2.79	3.40	[1,2]
Diazinon	-55.96	-32.80	4.06	3.80	[1,2]
Dichlorvos	-33.87	-19.80	2.47	1.40	[1,2]
Dimethoate	-80.37	-78.14	0.39	0.80	[1,2]
Dioxathion	-82.11	-56.76	4.44	4.30	[1,2]
Disulfoton	-65.43	-36.77	5.02	4.00	[1,2]
Ethion	-75.83	-51.91	4.19	5.10	[1,2]
Fenitrothion	-45.41	-51.61	-1.09	3.30	[1,2]
Fenthion	-47.53	-32.55	2.63	4.10	[1,5]
Fonofos	-50.87	-27.94	4.02	3.90	[1,2]
Malathion	-68.16	-46.74	3.75	2.40	[1,2]
Methyl Parathion	-67.41	-52.98	2.53	2.90	[1,2]
Monocrotophos	-44.76	-32.25	2.19	-0.20	[1,2]
Parathion	-71.69	-76.61	-0.86	3.80	[1,2]
Phorate	-53.42	-34.13	3.38	3.60	[1,2]
Phosalone	-56.74	-34.35	3.92	4.40	[1,2]
Temephos	-76.22	-57.67	3.25	6.00	[1,2]

Table S6. Calculated solvation free energy change of transfer from the gas phase to water phase (ΔGsolv(water) / kcal mol−1) and octanol phase (ΔGsolv(octanol) / kcal mol−1) under standard state conditions, and corresponding log KOW values of examined OPs pesticide set at B3LYP/DEF2-TZVP level of theory, with experimentally determined log P.

OPs	ΔGOctanol	ΔGWater	LogKo/w(PBE-def2-SVP)	LogPexp	Ref.
Acephate	-72.86	-78.42	-0.97	-0.80	[1,2]
Aspon	-71.90	-30.61	7.24	6.00	[1,2]
Carbophenothion	-75.45	-47.51	4.90	5.30	[1,2]
Chlorpyrifos	-52.55	-26.25	4.61	5.00	[1,3]
Coumaphos	-87.76	-71.30	2.88	4.50	[1,4]
Crufomate	-77.01	-62.27	2.58	3.40	[1,2]
Diazinon	-57.57	-34.95	3.97	3.80	[1,2]
Dichlorvos	-37.94	-25.05	2.26	1.40	[1,2]
Dimethoate	-83.94	-83.22	0.13	0.80	[1,2]
Dioxathion	-94.67	-63.57	5.45	4.30	[1,2]
Disulfoton	-67.22	-39.84	4.80	4.00	[1,2]
Ethion	-79.44	-56.00	4.11	5.10	[1,2]
Fenitrothion	-53.35	-39.56	2.42	3.30	[1,2]
Fenthion	-51.73	-28.18	4.13	4.10	[1,5]

Fonofos	-70.24	-49.28	3.67	3.90	[1,2]
Malathion	-71.84	-59.18	2.22	2.40	[1,2]
Methyl Parathion	-49.24	-37.01	2.14	2.90	[1,2]
Monocrotophos	-82.31	-89.82	-1.32	-0.20	[1,2]
Parathion	-57.80	-39.68	3.18	3.80	[1,2]
Phorate	-58.02	-34.28	4.16	3.60	[1,2]
Phosalone	-83.30	-66.05	3.02	4.40	[1,2]
Temephos	-82.73	-49.20	5.88	6.00	[1,2]

Table S7. Calculated solvation free energy change of transfer from the gas phase to water phase (ΔGsolv(water) / kcal mol−1) and octanol phase (ΔGsolv(octanol) / kcal mol−1) under standard state conditions, and corresponding log KOW values of examined OPs pesticide set at PBEh-3c level of theory, with experimentally determined log P.

OPs	ΔGOctanol	ΔGWater	LogKo/w(PBE-def2-SVP)	LogPexp	Ref.
Acephate	-73.41	-79.67	-1.10	-0.80	[1,2]
Aspon	-71.60	-30.59	7.19	6.00	[1,2]
Carbophenothion	-76.44	-48.52	4.89	5.30	[1,2]
Chlorpyrifos	-52.30	-24.82	4.82	5.00	[1,3]
Coumaphos	-84.41	-66.65	3.11	4.50	[1,4]
Crufomate	-78.83	-65.01	2.42	3.40	[1,2]
Diazinon	-59.19	-36.83	3.92	3.80	[1,2]
Dichlorvos	-38.86	-27.04	2.07	1.40	[1,2]
Dimethoate	-84.65	-84.08	0.10	0.80	[1,2]
Dioxathion	-95.93	-65.56	5.32	4.30	[1,2]
Disulfoton	-64.60	-37.30	4.78	4.00	[1,2]
Ethion	-79.36	-59.41	3.50	5.10	[1,2]
Fenitrothion	-51.12	-36.86	2.50	3.30	[1,2]
Fenthion	-57.36	-34.84	3.95	4.10	[1,5]
Fonofos	-75.01	-54.30	3.63	3.90	[1,2]
Malathion	-69.73	-59.31	1.83	2.40	[1,2]
Methyl Parathion	-48.09	-35.83	2.15	2.90	[1,2]
Monocrotophos	-80.41	-88.16	-1.36	-0.20	[1,2]
Parathion	-55.92	-36.65	3.38	3.80	[1,2]
Phorate	-57.95	-35.29	3.97	3.60	[1,2]
Phosalone	-82.56	-65.73	2.95	4.40	[1,2]
Temephos	-90.69	-57.81	5.76	6.00	[1,2]

Table S8. Calculated solvation free energy change of transfer from the gas phase to water phase (ΔGsolv(water) / kcal mol−1) and octanol phase (ΔGsolv(octanol) / kcal mol−1) under standard state conditions, and corresponding log KOW values of examined OPs pesticide set at PBE0/DEF2-SVP level of theory, with experimentally determined log P.

OPs	ΔGOctanol	ΔGWater	LogKo/w(PBE-def2-SVP)	LogPexp	Ref.
Acephate	-65.93	-70.47	-0.80	-0.80	[1,2]
Aspon	-67.43	-25.96	7.27	6.00	[1,2]
Carbophenothion	-69.79	-40.98	5.05	5.30	[1,2]
Chlorpyrifos	-49.70	-19.57	5.28	5.00	[1,3]
Coumaphos	-78.40	-59.84	3.25	4.50	[1,4]
Crufomate	-69.29	-53.92	2.70	3.40	[1,2]
Diazinon	-54.66	-31.39	4.08	3.80	[1,2]
Dichlorvos	-36.34	-24.04	2.16	1.40	[1,2]
Dimethoate	-72.38	-71.27	0.20	0.80	[1,2]
Dioxathion	-90.90	-59.79	5.45	4.30	[1,2]
Disulfoton	-60.43	-32.59	4.88	4.00	[1,2]
Ethion	-71.88	-53.56	3.21	5.10	[1,2]
Fenitrothion	-47.92	-33.59	2.51	3.30	[1,2]

Fenthion	-51.13	-27.91	4.07	4.10	[1,5]
Fonofos	-66.56	-44.61	3.85	3.90	[1,2]
Malathion	-64.96	-52.25	2.23	2.40	[1,2]
Methyl Parathion	-45.21	-32.85	2.17	2.90	[1,2]
Monocrotophos	-68.60	-73.85	-0.92	-0.20	[1,2]
Parathion	-51.32	-32.72	3.26	3.80	[1,2]
Phorate	-53.65	-28.83	4.35	3.60	[1,2]
Phosalone	-77.18	-59.76	3.05	4.40	[1,2]
Temephos	-80.94	-47.21	5.91	6.00	[1,2]

Table S9. Calculated solvation free energy change of transfer from the gas phase to water phase (ΔGsolv(water) / kcal mol−1) and octanol phase (ΔGsolv(octanol) / kcal mol−1) under standard state conditions, and corresponding log KOW values of examined OPs pesticide set at PBE0/DEF2-TZVP level of theory, with experimentally determined log P.

OPs	ΔGOctanol	ΔGWater	LogKo/w(PBE-def2-SVP)	LogPexp	Ref.
Acephate	-72.59	-79.66	-1.24	-0.80	[1,2]
Aspon	-69.65	-28.02	7.30	6.00	[1,2]
Carbophenothion	-75.71	-47.75	4.90	5.30	[1,2]
Chlorpyrifos	-50.70	-23.07	4.84	5.00	[1,3]
Coumaphos	-85.30	-67.85	3.06	4.50	[1,4]
Crufomate	-77.57	-62.73	2.60	3.40	[1,2]
Diazinon	-57.61	-34.65	4.02	3.80	[1,2]
Dichlorvos	-36.04	-22.73	2.33	1.40	[1,2]
Dimethoate	-84.26	-83.56	0.12	0.80	[1,2]
Dioxathion	-92.40	-60.60	5.57	4.30	[1,2]
Disulfoton	-66.93	-38.57	4.97	4.00	[1,2]
Ethion	-78.33	-54.64	4.15	5.10	[1,2]
Fenitrothion	-50.56	-35.84	2.58	3.30	[1,2]
Fenthion	-51.53	-28.04	4.12	4.10	[1,5]
Fonofos	-71.46	-50.64	3.65	3.90	[1,2]
Malathion	-69.33	-57.23	2.12	2.40	[1,2]
Methyl Parathion	-45.82	-32.60	2.32	2.90	[1,2]
Monocrotophos	-80.67	-87.61	-1.22	-0.20	[1,2]
Parathion	-54.73	-35.60	3.35	3.80	[1,2]
Phorate	-57.67	-33.20	4.29	3.60	[1,2]
Phosalone	-80.48	-63.22	3.03	4.40	[1,2]
Temephos	-80.47	-46.74	5.91	6.00	[1,2]

Table S10. Calculated solvation free energy change of transfer from the gas phase to water phase (ΔGsolv(water) / kcal mol−1) and octanol phase (ΔGsolv(octanol) / kcal mol−1) under standard state conditions, and corresponding log KOW values of examined OPs pesticide set at B97-3c level of theory, with experimentally determined log P.

OPs	ΔGOctanol	ΔGWater	LogKo/w(PBE-def2-SVP)	LogPexp	Ref.
Acephate	-71,71	-77,98	-1,10	-0.80	[1,2]
Aspon	-71,53	-28,94	7,47	6.00	[1,2]
Carbophenothion	-75,92	-48,07	4,88	5.30	[1,2]
Chlorpyrifos	-52,27	-24,44	4,88	5.00	[1,3]
Coumaphos	-85,91	-69,60	2,86	4.50	[1,4]
Crufomate	-76,47	-61,88	2,56	3.40	[1,2]
Diazinon	-58,11	-35,89	3,90	3.80	[1,2]
Dichlorvos	-36,49	-23,36	2,30	1.40	[1,2]
Dimethoate	-84,13	-83,37	0,13	0.80	[1,2]
Dioxathion	-96,20	-64,97	5,47	4.30	[1,2]
Disulfoton	-68,09	-40,43	4,85	4.00	[1,2]
Ethion	-79,53	-56,35	4,06	5.10	[1,2]

Fenitrothion	-51,06	-36,77	2,51	3.30	[1,2]
Fenthion	-53,90	-31,63	3,90	4.10	[1,5]
Fonofos	-71,67	-51,27	3,58	3.90	[1,2]
Malathion	-72,93	-59,51	2,35	2.40	[1,2]
Methyl Parathion	-47,69	-35,71	2,10	2.90	[1,2]
Monocrotophos	-79,05	-85,64	-1,16	-0.20	[1,2]
Parathion	-56,69	-38,86	3,13	3.80	[1,2]
Phorate	-59,63	-36,63	4,03	3.60	[1,2]
Phosalone	-81,61	-64,94	2,92	4.40	[1,2]
Temephos	-81,82	-49,06	5,74	6.00	[1,2]

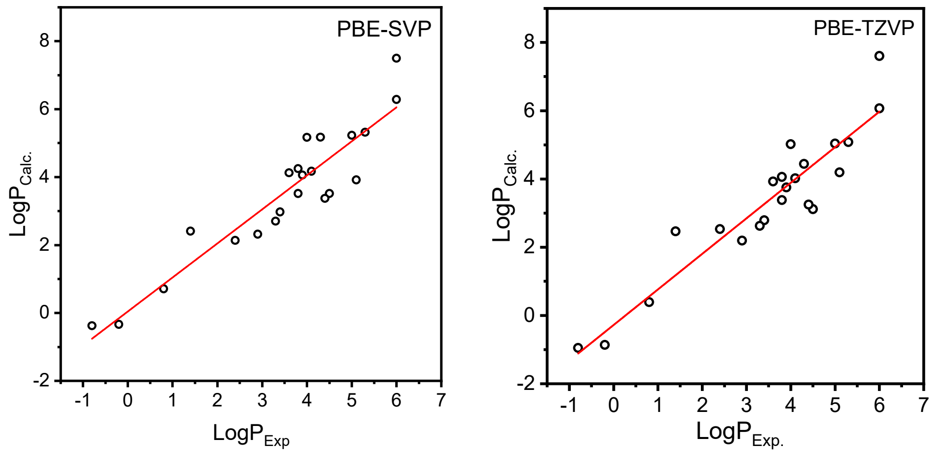

(a) (b)

Figure S1. Relation between experimental determined log P and LogPCalc calculated using PBE with two different basis - a) SVP and b) TZVP.

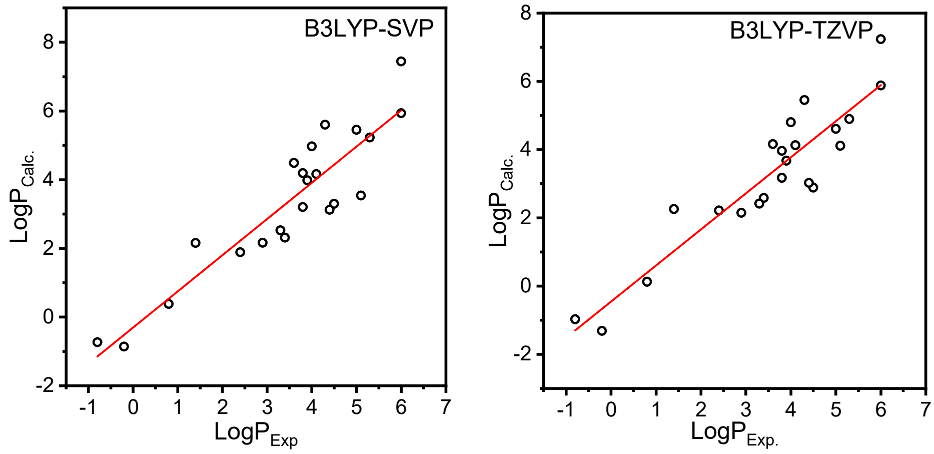

(a) (b)

Figure S2. Relation between experimental determined log P and LogPCalc calculated using B3LYP with two different basis - a) SVP and b) TZVP.

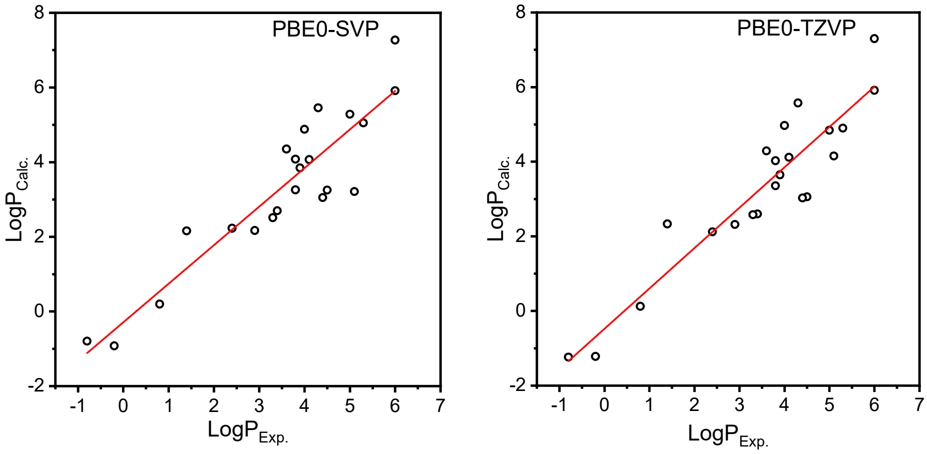

(a) (b)

Figure S3. Relation between experimental determined log P and $LogP_{Calc}$ calculated using PBE0 with two different basis - a) SVP and b) TZVP.

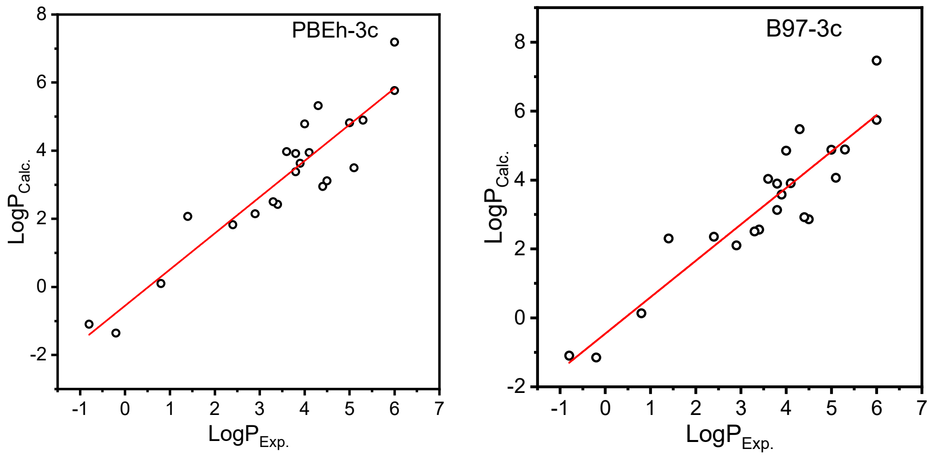

(a) (b)

Figure S4. Relation between experimental determined log P and $LogP_{Calc}$ calculated using Grimms methods - a) PBEh-3c and b) B97-3c.

References

1. Vlahovic, F.; Ivanovic, S.; Zlatar, M.; Gruden, M. Density functional theory calculation of lipophilicity for organophosphate type pesticides. Journal of the Serbian Chemical Society **2017**, 82, 1369–1378. doi:10.2298/JSC170725104V.
2. Martin, Y.C. Exploring QSAR: Hydrophobic, Electronic, and Steric Constants C. Hansch, A. Leo, and D. Hoekman. American Chemical Society, Washington, DC. 1995. Xix + 348 pp. 22 × 28.5 cm. Exploring QSAR: Fundamentals and Applications in Chemistry and Biology. C. Hansch and A. Leo. American Chemical Society, Washington, DC. 1995. Xvii + 557 pp. ISBN 0-8412-2993-7. Journal of Medicinal Chemistry **1996**, 39, 1189–1190, [https://doi.org/10.1021/jm950902o]. doi:10.1021/jm950902o.
3. Sangster, J. Octanol-Water Partition Coefficients of Simple Organic Compounds. Journal of Physical and Chemical Reference Data **1989**, 18, 1111–1229, [https://doi.org/10.1063/1.555833]. doi:10.1063/1.555833.
4. Garten, C.T.; Trabalka, J.R. Evaluation of models for predicting terrestrial food chain behavior of xenobiotics. Environmental Science & Technology **1983**, 17, 590–595, [https://doi.org/10.1021/es00116a006]. PMID: 22288703, doi:10.1021/es00116a006.
5. Bowman, B.; Sans,W. Determination of octanol-water partitioning coefficients (KOW) of 61 organophosphorus and carbamate insecticides and their relationship to respective water solubility (S) values. Journal of Environmental Science and Health, Part B **1983**, 18, 667–683, [https://doi.org/10.1080/03601238309372398]. doi:10.1080/03601238309372398.
